# Estimating a Stoichiometric Solid’s Gibbs Free Energy Model by Means of a Constrained Evolutionary Strategy

**DOI:** 10.3390/ma14020471

**Published:** 2021-01-19

**Authors:** Constantino Grau Turuelo, Sebastian Pinnau, Cornelia Breitkopf

**Affiliations:** Chair of Technical Thermodynamics, Technische Universität Dresden (TUD), Helmholtzstraße 14, 01069 Dresden, Germany; Sebastian.Pinnau@tu-dresden.de (S.P.); Cornelia.Breitkopf@tu-dresden.de (C.B.)

**Keywords:** constrained evolutionary strategy, thermodynamic model, stoichiometric solid model, NASA9, heat capacity, data dispersion, data fitting, vapor-solid equilibrium, salt hydrates, magnesium sulfate

## Abstract

Modeling of thermodynamic properties, like heat capacities for stoichiometric solids, includes the treatment of different sources of data which may be inconsistent and diverse. In this work, an approach based on the covariance matrix adaptation evolution strategy (CMA-ES) is proposed and described as an alternative method for data treatment and fitting with the support of data source dependent weight factors and physical constraints. This is applied to a Gibb’s Free Energy stoichiometric model for different magnesium sulfate hydrates by means of the NASA9 polynomial. Its behavior is proved by: (i) The comparison of the model to other standard methods for different heat capacity data, yielding a more plausible curve at high temperature ranges; (ii) the comparison of the fitted heat capacity values of MgSO_4_·7H_2_O against DSC measurements, resulting in a mean relative error of a 0.7% and a normalized root mean square deviation of 1.1%; and (iii) comparing the Van’t Hoff and proposed Stoichiometric model vapor-solid equilibrium curves to different literature data for MgSO_4_·7H_2_O, MgSO_4_·6H_2_O, and MgSO_4_·1H_2_O, resulting in similar equilibrium values, especially for MgSO_4_·7H_2_O and MgSO_4_·6H_2_O. The results show good agreement with the employed data and confirm this method as a viable alternative for fitting complex physically constrained data sets, while being a potential approach for automatic data fitting of substance data.

## 1. Introduction

Thermodynamic property estimation is an important step for establishing new innovation processes. In engineering and, especially, in material related disciplines, a lot of work has been applied into gathering and fitting data for describing different thermodynamic properties of the studied substances. Data points from different sources are not always consistent due to the employed methods, the experimental devices or even the composition of the sample.

Due to these discrepancies, it is important to develop and use methods more insensitive to data scattering as, a priori, it is impossible to discard or accept data directly by looking at the deviations if no uncertainty value is found. Furthermore, the data discarded on a first analysis could be still valid under certain conditions or confirmed when new data sets are added to the data pool.

**Figure 1:**
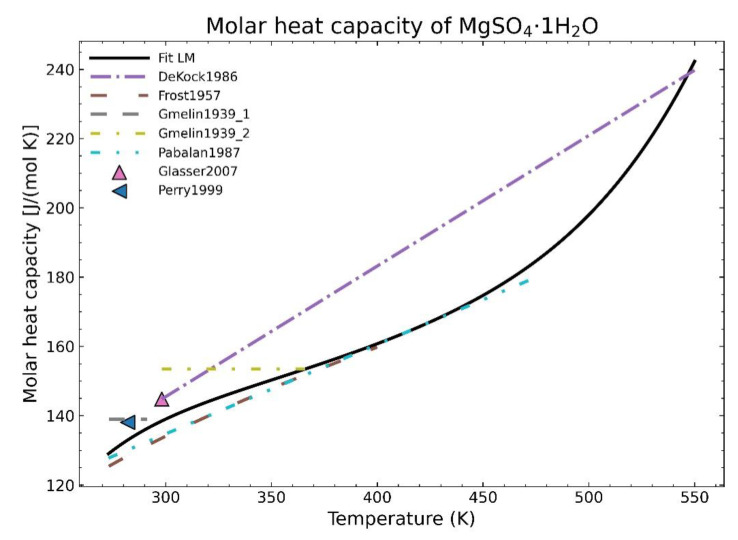
Fitting of the heat capacity for the substance MgSO_4_·1H_2_O using the Levenberg-Marquardt algorithm.

Typical fitting methods often rely on point densities. The procedure to model the curve tries to follow the path where most points are located without taking into account the overall picture or even trying to calculate a better curve where the overall least squares sum may be further minimized. Those methods, such as the Levenberg-Marquardt algorithm (LM) used in Scipy [1], are dependent mainly on local minima and frequently fail to preserve a proper physical behavior as shown in Figure 1.

The correct physical or thermodynamical description of the model is crucial to the goodness of the model. A precise statistical data fit that, on the other hand may lead to an incorrect phenomenological behavior, can hinder the practicality of the fitting process for further uses, especially at ranges where no data is to be found. In this work, utilizing a constrained evolutionary strategy (ES), an approach to find the global minima of the solution will be introduced, while preserving a physical meaning in the result due to the flexibility that such a method provides.

The mentioned ES, as will be shown later in this work, can be very effective for modeling the Gibbs’ Free Energy of stoichiometric solids. The Gibbs’ Free Energy *G* of an incompressible solid is given as a function of the enthalpy *H*, temperature *T*, and entropy *S* by the expression [2]:

*G* = *H* − *TS*. (1)

For incompressible solids, the change in properties due to volume or pressure changes are relatively small. So, for our purposes, the difference in enthalpy can be written as function of the heat capacity *C_p_* at constant pressure:

d*H* = *C_p_*d*T.* (2)

From the Second Law of Thermodynamics, the entropy can be defined as a function of the enthalpy which, according to (2), is dependent on the heat capacity and its temperature variation. Treating with incompressible substances guarantees that the variation of the pressure of the system is negligible:

*T*d*S* = d*H* − *V*d*p* (3)

d*S* = (1/*T*)d*H* = (*C_p_*/*T*)d*T.* (4)

Using this proved simplification, gathering temperature-dependent heat capacity data and combining it with the enthalpy and entropy of formation at standard conditions, the temperature-dependent enthalpies and entropies can be obtained. These expressions can be also used to check the correct thermodynamic description of the heat capacity fitting process.

In relation to the stoichiometric solid model, obtaining an accurate heat capacity curve is crucial for defining the Gibbs free energy of the solid. Depending on the substance of study, the amount of data can be scarce but, on the other hand, big datasets can present a large dispersion. For this reason, the use of more advanced fitting methods is increasingly important to deal with random or unexpected data distribution. Furthermore, it is an important prerequisite, that the fitting is physically feasible.

One important application of such fitted models is the modelling of the so-called phase change materials (PCMs). Phase Change Materials are promising substances for thermal energy storage applications, such as heat accumulators in power plants, building or industrial processes [3,4]. The ability to predict solid-liquid or solid-vapor phase equilibria of interesting substances, especially those that form eutectic mixtures is essential to understanding their thermal melting behavior, the working temperature ranges, and the compounds that are formed at different working conditions. To understand the behavior of the solid compounds or solid composition of a given binary mixture, a suitable and thermodynamically correct Gibbs’ free energy model must be built from the fitting of heat capacity, enthalpy, and entropy data. In this work, while the approach can be generally used, the analysis is focused on the MgSO_4_/H_2_O system as it forms different hydrates that can be applied in heating, ventilation and air conditioning systems (HVAC) [5].

In this context, the heat capacity data for MgSO_4_·1H_2_O (see Table 1 and Figure 1) is one of those examples where the values between different data points could deviate more than 20% and that could profit from new fitting methods.

Regarding the modeling, there exist different models to describe thermodynamic properties of stoichiometric solids. Some examples would be the Shomate equation [17] or the NASA polynomial [18]. In this article, the NASA-9 polynomial will be employed. The representation of this polynomial is through its adimensional form. Heat capacity data divided by the gas constant *R* are represented as a seventh order polynomial:

*c_p_*(*T*)/*R* = a_0_^−2^ + a_1_*T*^−1^ + a_2_ + a_3_*T* + a_4_*T*^2^ + a_5_*T*^3^ + a_6_*T*^4^, (5)

which after integration of (2) and (4) yields the two remaining parameters needed for a complete thermodynamic description:

*h*(*T*)/*RT* = −a_0_*T*^−2^ + a_1_ln(*T*)*T*^−1^ + a_2_ + a_3_*T*/2 + a_4_*T*^2^/3 + a_5_*T*^3^/4 + a_6_*T*^4^/5 + a_7_/*T* (6)

*s*(*T*)/*R* = −a_0_*T*^−2^/2 − a_1_*T*^−1^ + a_2_ln(*T*) + a_3_*T* + a_4_*T*^2^/2 + a_5_*T*^3^/3 + a_6_*T*^4^/4 + a_8_. (7)

The NASA9 polynomial is the latest addition to the thermodynamic database of the NIST. The difference with previous approaches is that the high order polynomial allowed the use of one continuous function for a wider temperature range validity [19]. The use of continuous functions, instead of previous piecewise approaches, is also beneficial for its further use in the convergence of phase equilibrium software due to its continuity. The files generated are also simpler to read from the programming perspective. A high order heat capacity curve allows also a better description of its integral to obtain the enthalpy and entropy data. The high order of this polynomial increases the flexibility of the fitting algorithm: A larger set of conditions/constraints, which reduce the degrees of freedom of the fitting equation, and different curve behaviors could be applied. This is important as the fitting algorithm introduced in the next section needs to be constrained to consider the following points:

- data density,
- dispersion and abundance or lack of data sets,
- automation of the fitting procedure to avoid subjective data selection,
- correct thermodynamic model and its constraints,
- finding global minima considering the above.

These points will be discussed during the following section after introducing the different assumptions for the fitting approach.

## 2. Materials and Methods

In this section, the different steps to create the proposed fitting process are explained in detail. These steps involve problems like different data densities, the automation of the fitting procedure or the definition of appropriate physical constraints.

### 2.1. Dealing with Different Data Densities: Weight Factors

The first step deals with the problem of data density. Literature data can be found in different ways: equations, experimental points, theoretical approaches, etc. Typical fitting approaches would tend to follow the area of higher data density, as shown in Figure 1. In order to avoid big data deviations depending on the region, it is important to first establish some filters by applying different weights to the datasets so that the selection is more robust for any employed fitting method.

The proposed weight factors are distributed into two groups: data source and equation type used in the researched literature. Every group is separated into 4 levels; the weight factor of every corresponding level would be the negative power of two of the level positions, starting with 0. Table 2 shows the given weight factors.

The proposed classification of data sources as weight factors with decreasing order is as follows:

1. Experimental: Data points collected from experimental devices as they are most likely to be precise and reproducible.
2. Mixed: A combination of experimental and theoretical approaches to obtain a given curve. For example, a fitted curve from a few experimental points. This is especially suitable when experimental points cannot be found but an equation from supposed experimental data is given.
3. Theoretical/Table: Data obtained through simulations or theoretical formulations derived from other kind of properties. Table means that the value is from known data collection tables.
4. Singular data: Singular points whose origin is unclear.

From the equation type perspective, the classification in weights following a decreasing order is:

1. Point: Just data points, they are often associated with experimental data and the combination of both gives the best weight possible.
2. Quadratic: Quadratic (or higher order) equation or curve used to fit the data.
3. Linear: the data comes in form of a linear temperature dependent fit.
4. Constant: a constant value given for a range of temperatures.
5. Not used: This extended flag has a weight of 0 and it is used to deactivate certain values for the fitting procedure without losing them entirely.

The overall weight factor is the multiplication of both weight factors. The highest weight would correspond to experimental points while the lowest one would be singular data (unclear sources) with constant values. However, they are still important if no other type of data is present. The weight factors for every *i*th data, *w_i,j_* for the *j*th data source and *w_i,k_* for the *k*th equation type, are then employed for the least squares residual to give the corresponding relative importance:

Σ_i_(*c_p_*_,*i*_^data^ − *c_p_*_,*i*_^fitted^)^2^*w_i_*_,*jk*_, (8)

where

*w_i,jk_* = *w_i,j_ w_i,k_* (9)

In order to see the effect of such weight factors, data for anhydrous magnesium sulfate will be employed. The data gathered to fit the corresponding NASA9 polynomial for the salt are described in Table 3.

**Figure 2:**
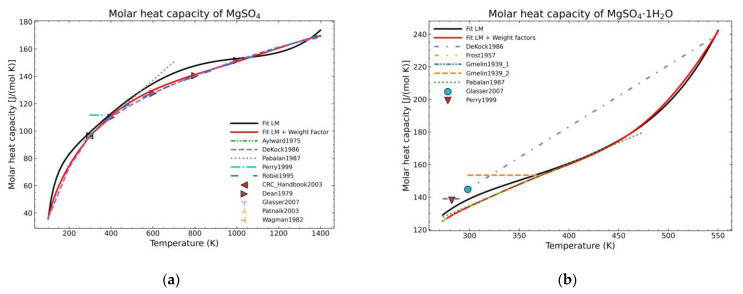
Comparison of fit results from a standard LM-algorithm and the addition of weight factors (red) for: (**a**) Anhydrous magnesium sulfate; (**b**) magnesium sulfate monohydrate.

For comparison purposes, two different forms of appearance for MgSO_4_ are shown in Figure 2. The graph corresponding to the pure salt (Figure 2a) shows an improvement regarding the expected shape of the curve (red line) as, due to the defined weight factors, the algorithm tends to follow the most trusting dataset within the defined parameters. However, using exclusively this approach is not effective, especially for areas where only one set of data, which is much deviated with respect to the previous fitted points, exists, as shown in the Figure 2b for temperatures higher than 470 K. On the other hand, even in this unfavorable situation, there is an improvement for the lower temperature points.

Another problem for the fitting of those heat capacity data is that the curve tends to go to the infinite, when the temperature is extrapolated above the temperature range covered by the gathered data. However, the extrapolation of the heat capacity curve to lower and higher temperature ranges is needed for phase equilibria calculations.

To improve the fitting outcome automatically without subjective influence, the fitting method itself should be revamped and that is where the evolutionary strategy comes into play.

### 2.2. Evolutionary Strategy Method

In this section, the strategy used to fit data in an improved way will be briefly explained. The employed method is based on the covariance matrix evolutionary strategy (CMA-ES), developed by Hansen and Ostermeier [25,26], as implemented in the DEAP computation framework, which can be used with the Python programming language [27]. Evolutionary strategies are stochastic methods with no derivation in the algorithm. This provides a more robust and stable solution, especially for non-linear optimization problems or those that are poorly conditioned.

The basis of evolutionary algorithms relies on genetic evolution through the selection of a new average distribution value (recombination) and the addition of a random vector, which is a disturbance with an averaged zero value (mutation). Individuals, which are solution sets, are generated each iteration after the mutation step. The generation of individuals is led by the minimization/maximization of the value of the objective function. In every step update, the paired dependencies between variables and the distribution of the individuals are represented by a covariance matrix. The next recombination step or the finding of the new average distribution value is done by the ranking of the individuals after evaluating the objective function with them. The recombination should follow the direction of the best ranked individuals. Due to the nature of the CMA-ES, unlike most classical methods, the character of the main objective function is not so important.

**Figure 3:**
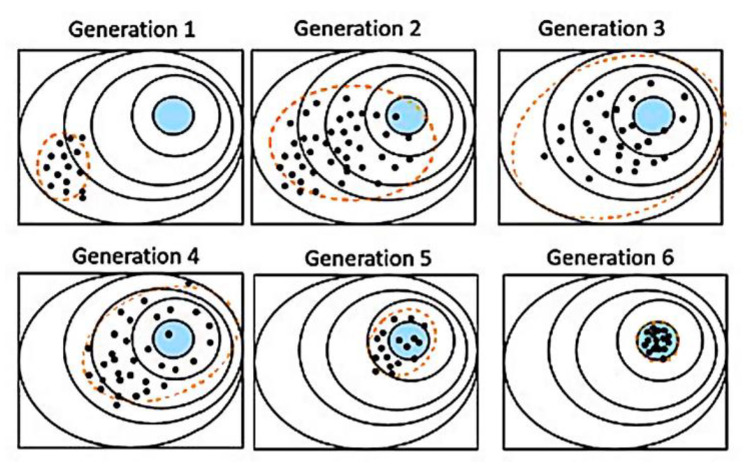
Example of two-dimensional spherical optimization problem. Points are individuals, the blue area is the solution area, and the dashed line is the dispersion calculated with the covariance matrix. Generation 1 shows the initial random distribution. Generations 2–3 show the population moving towards the detected favorable direction of the solution due to the ranking of the individuals. Higher random dispersion is expected as the algorithm looks for other possible minima in the vicinity by “mutation”. In generations 4–6, after the individuals are sufficiently dispersed, with the information of the covariance matrix, the global minimum is pinpointed and all the individuals, regardless of their initial position, converge to the final solution area.

Figure 3 illustrates a typical fitting process of a two-dimensional spherical optimization problem. On this simple problem, the population was concentrated on the global optimum after several iterations.

The CMA-ES algorithm is calculated by sampling a multi-variable normal distribution [26]. For the iteration *g* = 0, 1, 2, 3… the set of searched variables ***x*** is calculated through:

***x****_k_*^(*g* + 1)^ ∼ 𝒩(***m***^(*g*)^, (*σ*^(*g*)^)^2^***C***^(*g*)^) for *k* = 1,…, *λ*, (10)

where *λ* is the population size (number of calculated individuals), ***m*** is the mean value of the search distribution, *σ* is the so-called overall standard deviation or step size and ***C*** is the covariance matrix. There are different principles to adapt the searching parameters:

1. The principle of maximum probability: The average distribution value is updated to maximize the likelihood that the previous most successful individuals are closer to the final solution. This is the selection and recombination step. The mean of the search distribution comes from the selection of the most relevant *μ* selected points from the sample. Writing the weight (not the same as the weight factors from the previous subpart) as *ω_i_* with the condition that:

Σ*_i_ω_i_* = 1 for *i* = 1,…, *μ* and *μ* ≤ *λ*, (11)

the mean of the search distribution is then:

***m***^(*g* + 1)^ = Σ*_i_ω_i_**x**_i:__λ_*^(*g* + 1)^ for *i* = 1,…, *μ* (12)

If the setting is *ω_i_* = 1/*μ*, then, the mean of the search distribution is purely the mean value of the selected points. The recombination step is implicitly defined by the modification of the weight coefficients.

2. Two types of temporal evolution of the average statistical distribution of the strategy are recorded. These paths contain significant information about the correlation between successive steps. In particular, the algorithm is effective if there is a large positive evolution in successive steps in the same direction. To do that, the covariance matrix, within the weighted selection mechanism is calculated with the following expression:

***C****_μ_*^(*g* + 1)^ = Σ*_i_ω_i_* (***x****_i:__λ_*^(*g* + 1)^ − ***m***^(*g*)^) (***x****_i:__λ_*^(*g* + 1)^ − ***m***^(*g*)^)^T^ (13)

To allow a higher weight of the recent operations to the covariance matrix, a new factor 0 < *c*_cov_ ≤ 1 is introduced as learning rate (exponential smoothing), where with 0, there is no learning and 1 takes no older steps into consideration. Being ***C***^(0)^ = I (identity matrix), the recalculation of the covariance matrix is:

***C***^(*g*+1)^ = (1 − *c*_cov_) ***C***^(*g*)^ + *c*_cov_***C***_μ_^(*g*+1)^/(*σ*^(*g*)^)^2^ (14)

After that, the tracking of the evolution path is needed to accelerate following dispersion steps. Defining ***p***_c_ as the evolution path, with ***p***_c_^(0)^ = 0 and a learning rate factor, *c*_c_:

***p***_c_^(*g*+1)^ = (1 − *c*_c_) ***p***^(*g*)^ + (*c*_c_ (2 − *c*_c_) *μ*_eff_)^1/2^ (***m***^(*g*+1)^ − ***m***^(*g*)^)/*σ*^(*g*)^, (15)

where

*μ*_eff_ = (Σ*_i_ω_i_*^2^)^−1^ (16)

This is called the cumulation. Combining all the previous steps, the final expression for the update of the covariance matrix is achieved:

***C***^(*g* + 1)^ = (1 − *c*_cov_) ***C***^(*g*)^ + (*c*_cov_/*μ*_eff_) ***p***_c_^(*g* + 1)^ (***p***_c_^(*g* + 1)^)^T^ + *c*_cov_(1 − 1/*μ*_eff_) ***C****_μ_*^(*g* + 1)^/(*σ*^(*g*)^)^2^. (17)

The second temporal evolution is a step-by-step control which studies the sequential movement of the orthogonal distribution of the average distribution value, preventing premature convergence before it really occurs, while still achieving a proper convergence speed. This is done by the automatic control of the step size. Firstly, a conjugate evolution path in comparison to (15) is built:

***p***_σ_^(*g*+1)^ = (1 − *c_σ_*) ***p***^(*g*)^ + (*c_σ_* (2 − *c_σ_*) *μ*_eff_)^1/2^ (***C***^(*g*)^)^−1/2^ (***m***^(*g*+1)^ − ***m***^(*g*)^)/*σ*^(*g*)^. (18)

After that, the evolution path is compared to an expected distance given by a random distribution, measured by the Euclidean norm (E) of a distributed random vector, (0, **I**). Defining d_σ_ as a damping parameter which should be close to one, the step size is calculated as follows:

*σ*^(*g*+1)^ = *σ*^(*g*)^ exp(*c_σ_*/*d_σ_* ((||***p****_σ_*^(*g*+1)^||/E||𝒩(0, **I**)||) − 1)). (19)

As with the previous set of equations, the algorithm cycle consists of three main parts: (1) sampling of new solutions, (2) reorder of selected solutions according to their suitability, (3) updating of internal state variables on the basis of the reordered samples as shown in the previous equations. In this work, the DEAP implementation is used without further internal modifications so that only the population size and the number of iterations are controlled. The initial distribution can be also changed, but no systematic improvement was observed.

In this method, due to the application of the mutation (perturbation of the best-found solution) and the possibility of following more than one convergence direction, it is more likely to find a solution closer to a global minimum instead of converging prematurely to possible existing local minima. Furthermore, the lack of derivatives in the algorithm improves the fitting stability. However, the number of iterations is usually larger than other more common fitting algorithms. On the other hand, another advantage is the possibility of implementing more complex conditions in DEAP. Those conditions will be discussed in the following subsection. The results of the method will be discussed in the results section.

### 2.3. Definition of Constraints: Debye Model

In order to have a realistic heat capacity curve, a suitable physically plausible model should be utilized through the definition of constraints to improve the usability of the fitted model. For this work, the heat capacity Debye model will be introduced to define the shape of the heat capacity curve [28]. This approach has two purposes: (1) The right prediction of the heat capacity values outside the temperature range where data is available to influence the correctness of the range with existing data; (2) to help to choose the right trend of the existing data points regardless of the point density as they can have very different slopes and dispersions.

**Figure 4:**
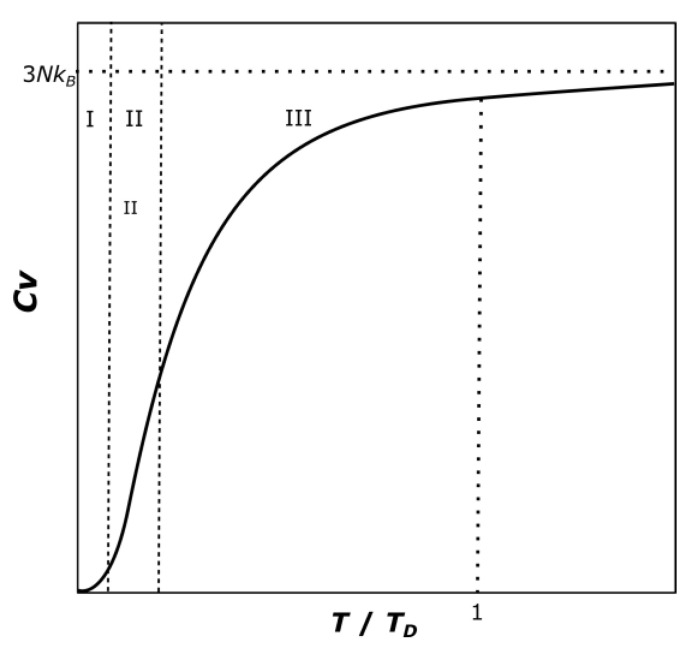
Qualitative curve of the Debye model, the three regions indicate the application of the different established conditions.

The Debye Model estimates the heat capacity of a solid through the phonon contribution. The phononic structure is directly related to the thermodynamic properties of solids [29]. The phonon density of states determines the thermal properties of the crystal. In the case of the calculation of the thermal conductivity, it is mainly achieved by the low frequency part of the distribution, while the calculation of the heat capacity is dominated by the high-frequency region. The assumptions made by Debye (constant speed of sound) causes a good prediction at low and high temperature ranges but incorrect results at intermediate temperatures [30]. A typical Debye model curve can be observed in Figure 4.

In this model, there are two temperature regimes which are dependent on the Debye temperature *T*_D_ of the solid. *T*_D_ is defined as:

*T*_D_ = *hυ*/*k*_B_ (20)

where *h* is the Planck constant, *υ* is the Debye frequency (typically the maximum frequency of the vibrations of the atoms of the solid), and *k*_B_ is the Boltzmann constant. This value can range from dozens to thousands of Kelvin, depending on the solid [29].

The Debye model at low temperature range, i.e., for temperatures much lower than the Debye Temperature *T*_D_, predicts that the shape of the curve is proportional to *T*^3^. This would correlate to the region I in Figure 4. The value of the heat capacity at constant volume in that region is:

*C_v_*/*Nk*_B_ = 12π^4^/5 (*T*/*T*_D_)^3^ (21)

where *N* is the number of atoms in the solid. In this work, to simplify the relation and make it more general for any thermodynamic model, the first constraint is defined at a very small low temperature range, which is chosen arbitrarily as between 1–5 K. Following the Debye model, the heat capacity must have a positive increasing slope:

d^2^*C_p_*/d*T*^2^ ≥ 0 for 1 K ≤ *T* ≤ 5 K (22)

At high temperature ranges, for temperatures much higher than the Debye temperature, the heat capacity should approach an asymptotic value provided by the Dulong-Petit Law [31], which is quite accurate in most cases, but it does not consider all possible effects:

*C_v_*/*Nk*_B_ = 3 (23)

This means that the curve at intermediate and high temperature levels should have a decreasing slope. This defines the second constraint. Between the temperatures of 50–2000 K (region III in Figure 4), the condition used will be:

d^2^*C_p_*/d*T*^2^ ≤ 0 for 50 K ≤ *T* ≤ 2000 K (24)

The value 2000 K was arbitrarily chosen as a sufficiently large temperature to check the validity of the Debye model even over the pure compound existence range.

The last constraint can be obtained by looking at the shape of the typical heat capacity curve (Figure 4), the heat capacity increases as the temperature increases. The curve is monotonically ascending. This can be used as the third necessary condition:

d*C_p_*/d*T* ≥ 0 for 1 K ≤ *T* ≤ 2000 K (25)

However, the second derivative is not defined in the temperature range between 5 K and 50 K (region II in Figure 4). This is done to let the NASA polynomial equation adapt itself between the two given conditions, giving the fitting process a higher flexibility and not to force an unsolvable constraint.

The next step is to add these conditions into the objective function that must be minimized. To comply with this, the addition of penalties becomes necessary. In this work, the suggested approach is to force these conditions in a range of temperatures corresponding to 1 K–2000 K. A set of, minimum, 100 temperature points should be checked to prove if the three conditions are fulfilled in the suggested range. For every point and condition not fulfilled, a fixed penalty value will be added to the residual. The CMA-ES algorithm should detect in which direction the numbers of points, which fulfill all conditions, are higher and improve the convergence rate when checking the average distribution value.

Penalty expressions can be chosen depending on the data and the computed unconstrained residual, i.e., the values of the function that must be minimized, based on (8). Once the first and last residual are computed, the penalty values can be assigned. Those penalty values should be always of one or more orders of magnitude lower than the first residual computed without any condition and, at least, one or more orders of magnitude higher that the last residual computed without conditions. This is done to discard very aggressive penalty values, which could force a premature convergence in local minima and to give the user of the algorithm a first estimation of which values should be used.

The conditions, counting also with a positive heat capacity value, are then summarized in the following set of expressions where every constraint corresponds to the generation of a respective penalty (*pty*). This is evaluated in a set of l-points *l* = [1, 2000] with chosen evaluation steps (100 by default):

if *c_p_*^fitted^(*l*) ≤ 0, a value *pty*_0,*l*_ is added for 1 ≤ *l* ≤ 2000 if d *c_p_*^fitted^(*l*)/d*T* ≤ 0, a value *pty*_1,*l*_ is added for 1 ≤ *l* ≤ 2000 if d^2^*c_p_*^fitted^(*l*)/d*T*^2^ ≤ 0, a value *pty*_2,*l*_ is added for 50 ≤ *l* ≤ 2000 if d^2^*c_p_*^fitted^(*l*)/d*T*^2^ ≤ 0, a value *pty*_3,*l*_ is added for 1 ≤ *l* ≤ 5 (26)

The new minimization function, considering the previous definition, is then:

Σ*_i_*(*c_p,i_*^data^ − *c_p,i_*^fitted^)^2^*w_i,jk_* + Σ*_l_*(*pty*_0,*l*_ + *pty*_1,*l*_ + *pty*_2,*l*_ + *pty*_3,*l*_) (27)

The residual would then scale with the number of points that do not fulfill the said conditions which helps the convergence of the CMA-ES method by showing which individuals (set of solutions) present fewer invalid points, forcing the algorithm to follow those.

With the control of the residual, that is to say, by checking the last residual value given by (27), the user can observe if all the conditions were fulfilled during the fitting process or, on the contrary, if new parameters or further iterations are necessary. If the final residual value is of the order of magnitude of one of the added penalties, the penalty value should be tuned again to find better solutions. For instance, if a penalty value of 10^4^ is defined and the final residual is 1.10003 × 10^5^, 11 points did not satisfy the predefined condition and the fitting procedure failed. In other words, other penalty values should be used to improve the final result but without hiding their effects.

With the suggested method presented in this work, apart from the penalty values which must be chosen per substance, there are three further tunable parameters from the DEAP CMA-ES implementation: (1) sigma which is the initial dispersion of the first random chosen populations; (2) lambda, which is the number of individuals (possible solutions) of the population; and (3) number of iterations. Sigma can influence how fast the solution is obtained and has no systematic behavior. The values used in this work were tuned for the fitted data; it should not have an impact in the end result, only in the iterations needed. Lambda is usually chosen as a multiplier from the number of fitting parameters. The higher lambda is, the lower the number of iterations is required to achieve convergence. However, it can be slower than a lower lambda value with higher number of iterations.

The previous description of the algorithm was centered on the heat capacity. For the description of the free energy of the stoichiometric solid, the enthalpy and entropy must be also calculated. However, they are easier to calculate as the shape of the enthalpy/entropy curve is implicitly defined with the heat capacity data. In other words, the correct shape of the heat capacity curve will also yield correct enthalpy and entropy curves, showing the importance of having a correct thermodynamic definition from the beginning of the fitting process. With the heat capacity data, 7 of the 9 NASA9 parameters can be calculated. It is worth noting that a decrease in the degrees of freedom of the fitted equation occurs with the definition of the previous four constraints. The Shomate equation would be insufficient for fitting a wide temperature range equation with the mentioned thermodynamic constraints. It would be also difficult to fulfill all conditions with a NASA7 polynomial (5 parameters to fit heat capacity data). For this reason, the use of the NASA9 for the defined approach would be optimal. The other two parameters of the NASA9 polynomial come from the fitting of the standard enthalpy/entropy of formation data by the use of DEAP without penalty factors. From there, the Gibbs’ free energy data is easily obtainable.

The fitting procedure is considered successful if no significant residual change is noticed during the last simulation steps and the value is lower than any of the penalty functions given unless no better solution was found.

## 3. Results and Discussion

In this section, the presented algorithm will be used to calculate the thermodynamic properties of different hydrate levels of MgSO_4_. This is a test case to see how the model behaves with different data dispersion. After that, the obtained heat capacity curve of MgSO_4_·7H_2_O will be compared to differential scanning calorimeter (DSC) data taken from internal experimental testing. Finally, an application of the Gibbs’ free energy model will be presented, using the equilibrium vapor pressure of the hydrates in comparison to the Van’t Hoff formulation.

### 3.1. Computation of NASA9 Polynomial for Different Hydrates of Magnesium Sulfate

Before fitting the data, it is necessary to do an intensive work of data gathering. In previous sections, the source of data for the anhydrous form of magnesium sulfate (Table 3) and the monohydrate form (Table 1) were presented. Table 4 shows the data source of other hydration levels, namely 2, 4, 5, 6, and 7, used in this work.

For practical purposes, the penalty functions were optimized for all hydrate levels at the same time, as this method is very useful to automatize the calculations and obtain all the hydration levels with unified parameters. Table 5 shows the parameters chosen for the penalty factors after performing a first fitting process without penalty factors.

Table 6 shows the fitting parameters of the DEAP CMA-ES along the final residuals of the fitting process. The population is chosen so that the number of individuals is 45 times the number of parameters which must be found. The iterations are manually selected depending on the convergence rate and the change of the residual is inspected to see if there are big changes in the last steps. Comparing the penalty factors of Table 5 and the residuals of the Table 6, it is visible that the conditions given are completely fulfilled as the residuals are in all cases lower than the penalty factors. If one point does not fulfill the condition, the additive character of the penalties would cause the residual to be larger than the penalty factor associated to one of the defined conditions.

As mentioned during the definition of the constraints, the calculation of extended temperature ranges, even over the pure substance existence range, is important for the calculation of phase equilibria. If there is a combination of temperature-pressure-composition whose mixture of substances is unknown (for instance, solid-solid. solid-liquid, or solid-vapor) due to the lack of data or impossibility of determining them experimentally, the mixture that would reach the minimum Gibbs free energy would be the existent mixture of substances at those conditions. This can only be theoretically calculated if the employed Gibbs free energy model of the compounds can be correctly defined on those extended points. In this work, as it could occur with some unknown mixture of substances, the existence boundaries will be assumed to be unknown in order not to influence the final result, as well as treating the model as a predictive model for phase equilibria calculation.

**Figure 5:**
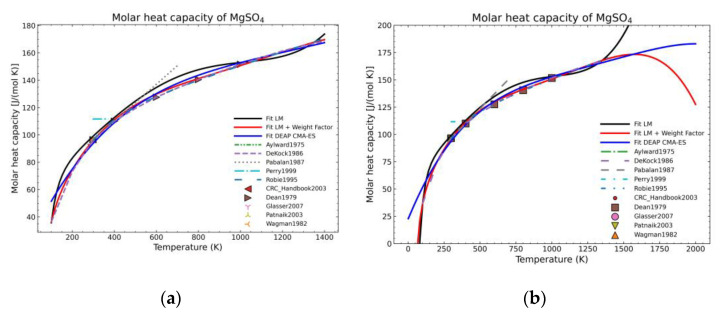
Comparison of the different fit results of MgSO_4_ showing: (**a**) literature points region; (**b**) extended range until 2000 K.

**Figure 6:**
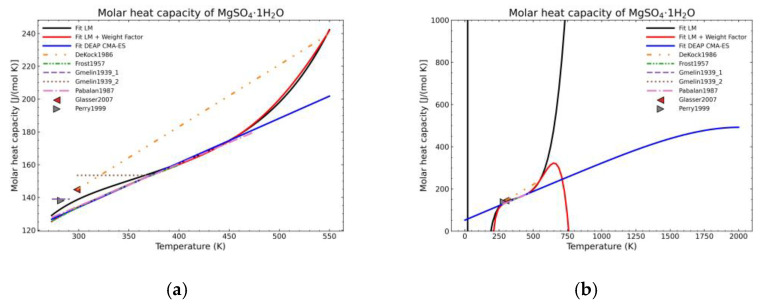
Comparison of the different fit results of MgSO_4_·1H_2_O showing: (**a**) literature points region; (**b**) extended range until 2000 K.

**Figure 7:**
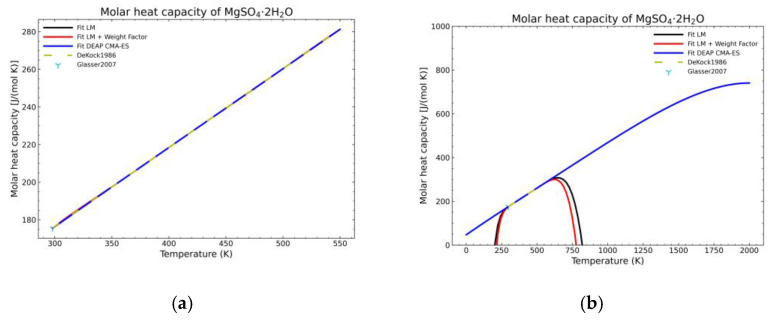
Comparison of the different fit results of MgSO_4_·2H_2_O showing: (**a**) literature points region; (**b**) extended range until 2000 K.

**Figure 8:**
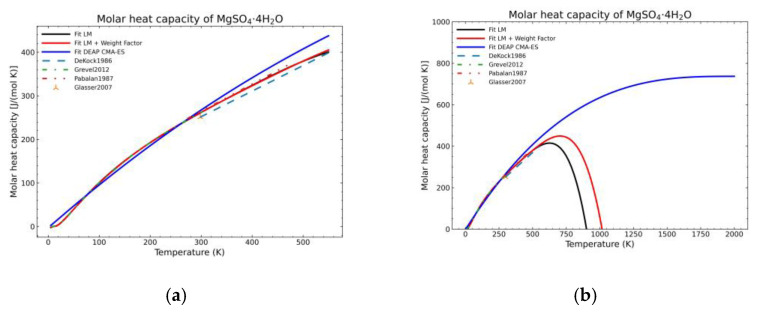
Comparison of the different fit results of MgSO_4_·4H_2_O showing: (**a**) literature points region; (**b**) extended range until 2000 K.

**Figure 9:**
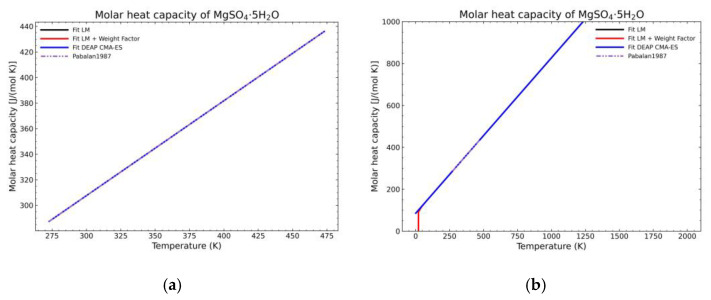
Comparison of the different fit results of MgSO_4_·5H_2_O showing: (**a**) literature points region; (**b**) extended range until 2000 K.

**Figure 10:**
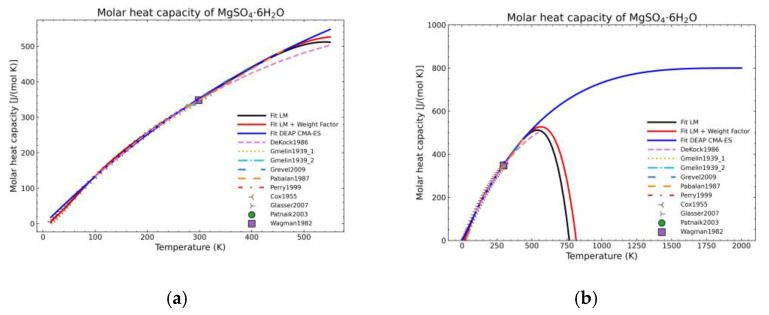
Comparison of the different fit results of MgSO_4_·6H_2_O showing: (**a**) literature points area; (**b**) extended range until 2000 K.

**Figure 11:**
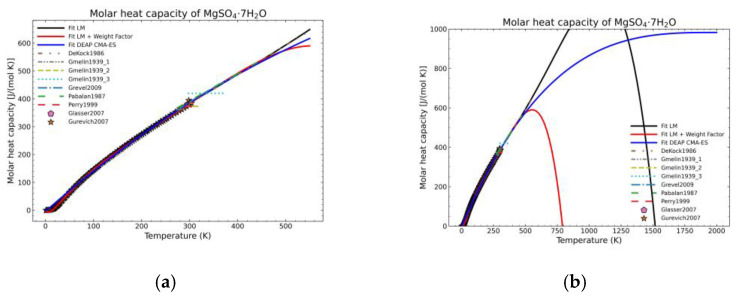
Comparison of the different fit results of MgSO_4_·7H_2_O showing: (**a**) literature points region; (**b**) extended range until 2000 K.

Figure 5, Figure 6, Figure 7, Figure 8, Figure 9, Figure 10 and Figure 11 show the fitting results in comparison with the LM method and LM with the proposed weight factors. While in some cases, the fitting procedures are similar in the literature points region, it changes drastically when the temperatures are extended beyond literature temperature points. The CMA-ES method is the only option retaining a thermodynamic feasible model at a very large temperature range, which also affects the fitting procedure at the region where data exist. At the same time, in substances whose literature points are difficult to fit due to its variability, the CMA-ES method provides a more consistent solution such as the one shown in Figure 6. This advantage is not isolated to heat capacities but applicable too to any fitting procedure where sufficient constraints can be defined with different data dispersion.

**Figure 12:**
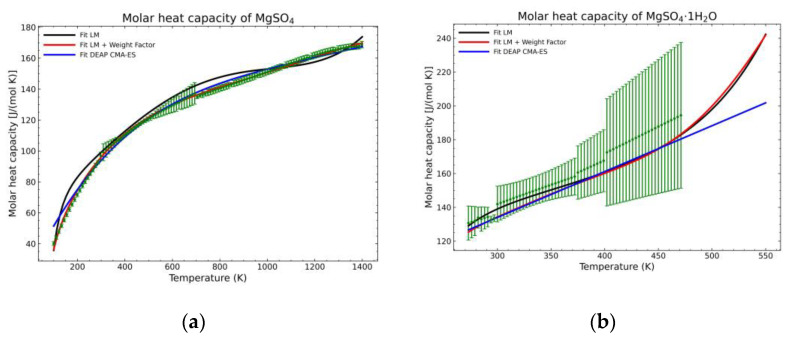
Comparison of the different fit results, showing the literature data in green (mean of the values) with the 95% confidence interval boundaries of: (**a**) MgSO_4_; (**b**) MgSO_4_·1H_2_O.

**Figure 13:**
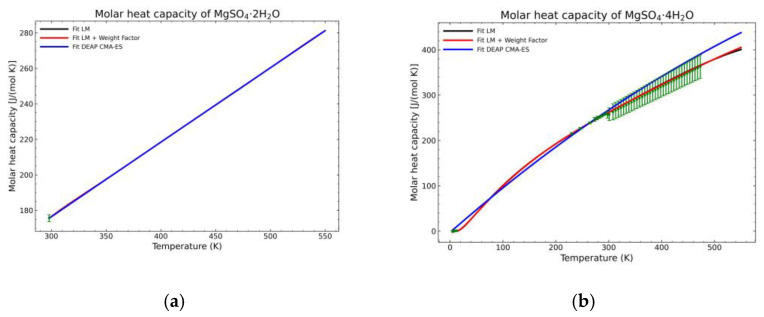
Comparison of the different fit results, showing the literature data in green (mean of the values) with the 95% confidence interval boundaries of: (**a**) MgSO_4_·2H_2_O; (**b**) MgSO_4_·4H_2_O.

**Figure 14:**
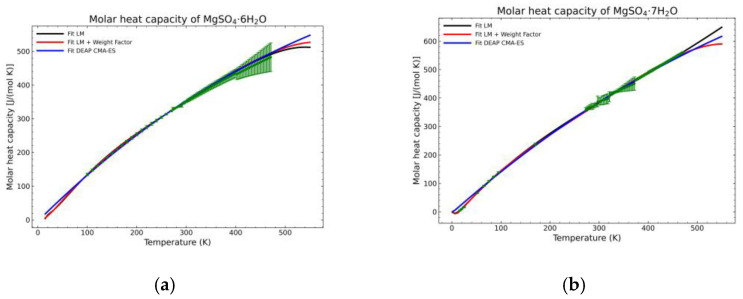
Comparison of the different fit results, showing the literature data in green (mean of the values) with the 95% confidence interval boundaries of: (**a**) MgSO_4_·6H_2_O; (**b**) MgSO_4_·7H_2_O.

A statistic overview of the different fitting methods with respect to the obtained data is shown in Figure 12, Figure 13 and Figure 14. The green points are the mean values of the gathered literature values while the error bars show the 95% confidence interval. These error bars are very dependent on the amount of data. If no more than two points are available for a temperature point, the confidence interval cannot be calculated as they depend on the standard error of the sampled population. For this reason, the pentahydrate form is not shown, as the amount of data was not sufficient to build the confidence interval. The standard error (*SE*) of the dataset at every temperature value, being *SD* the standard deviation of the sample with *n* points, is calculated as:

*SE* = *SD*/(*n*)^1/2^ (28)

The error bars would correspond to a 95% confidence interval (*CI*), defined as:

*CI(95%)* = ± 1.96 *SE* (29)

An error study was also performed to evaluate the different methods’ results with respect to the literature data. This should show if a correct thermodynamic definition could provide worse, similar, or better results within the literature temperature data range. In general, for a parameter *y* and population *n*, the two calculated statistical deviations will be the mean relative error (*MRE*) and the normalized root mean square deviation (*NRMSD*). *MRE* is defined as:

*MRE* = 1/*n*[Σ*_i_*(|*y_i_*^data^ − *y_i_*^fitted^|/*y_i_*^data^)] (30)

The *NRMSD* is calculated as:

*NRMSD* = |[Σ*_i_*(*y_i_*^data^ − *y_i_*^fitted^)^2^/*n*]^1/2^/[Σ*_i_*(*y_i_*^data^)/*n*]| (31)

Recalling the results from Figure 5, Figure 6, Figure 7, Figure 8, Figure 9, Figure 10 and Figure 11, in most fitted substances, the proposed CMA-ES method was showing a better curve shape, from the Thermodynamics perspective, in comparison to the other methods. On the other hand, Table 7, which shows the results of the statistical calculations, the CMA-ES method gives similar results, although slightly worse, in comparison to the other methods in the literature temperature range. In general, the CMA-ES method provides a much better thermodynamic description, especially beyond the data temperature range, and a curve shape which resembles the Debye model at a slight statistical penalty. For instance, one of the worst statistical results of the new proposed method is the fitted data of MgSO_4_·4H_2_O. However, when inspecting Figure 8, just after the data at the highest temperature, the heat capacity curve fitted with the LM method would just fall off, which is thermodynamically incorrect. This would be a source of errors when using the LM-obtained Gibbs free energy model to calculate phase equilibria beyond that temperature data range. In other cases, like in Figure 6 (monohydrate form), within the data range, the only thermodynamically correct curve shape is given by the CMA-ES method. While the constraints of the CMA-ES method are beneficial for the shape of the curve and its use in more complex phenomena, they are statistically not so favored.

**Figure 15:**
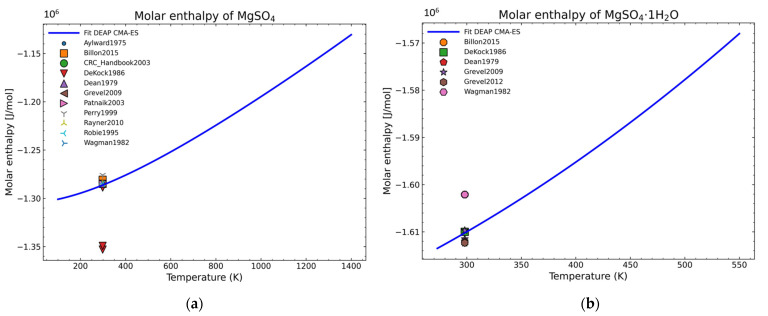
Fitted results of the enthalpy curve in the literature points region for: (**a**) MgSO_4_; (**b**) MgSO_4_·1H_2_O.

**Figure 16:**
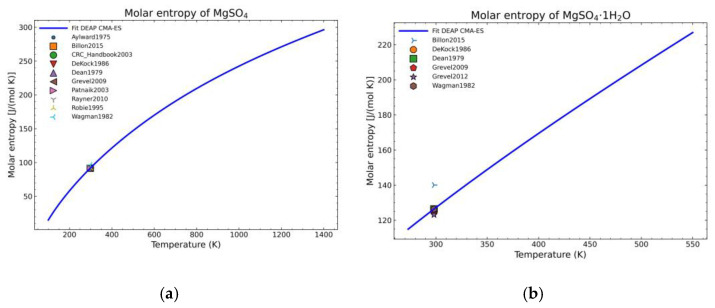
Fitted results of the entropy curve in the literature points region for: (**a**) MgSO_4_; (**b**) MgSO_4_·1H_2_O.

The fitting procedure for getting the heat capacity yields 7 of the 9 NASA9 parameters. For the remaining NASA9 polynomial parameters, any of the weighted procedures would yield the same result as only one extra point is fitted for the enthalpy and another one for the entropy. For that, the gathered data of the standard enthalpy of formation and standard entropy of formation are used. No further conditions are necessary as the heat capacity curve implicitly defines the shape of the enthalpy and entropy curve. The fitting parameters employed for the CMA-ES and the calculation of the remaining NASA9 parameters are displayed on Table 8. In order to give a better visualization of the fitted enthalpy and entropy results, Figure 15 and Figure 16 show the fitted results and literature values of MgSO_4_ and MgSO_4_·1H_2_O as examples.

**Figure 17:**
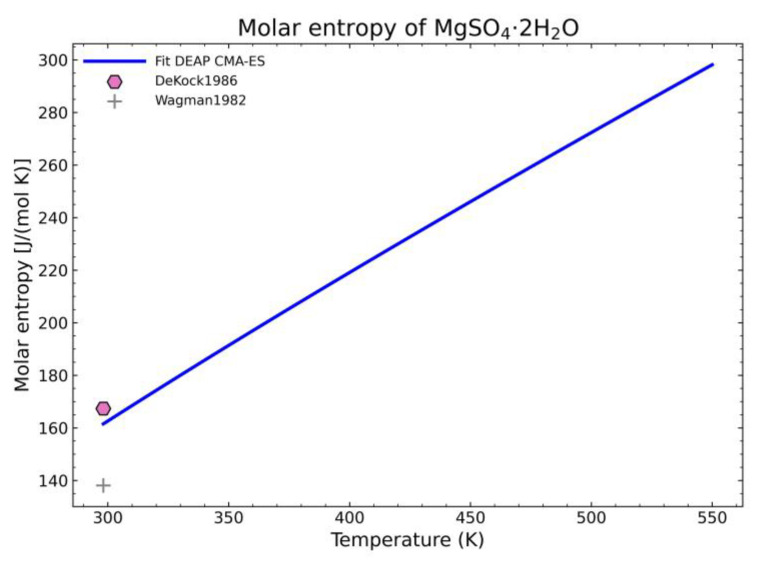
Fitted results of the entropy curve in the literature points region for MgSO_4_·2H_2_O.

The enthalpy and entropy data were also statistically analyzed. The results are gathered in Table 9, which are within the expected margin of error except for the error of the entropy values of MgSO_4_·2H_2_O. This error value is too large. However, when inspecting the corresponding fitted diagram (Figure 17), this is caused by the low number of points and the suitability of one point over the other with the already fitted parameters (8 of 9 NASA9 parameters are determined before fitting the entropy curve) so it is an unavoidable error with the current dataset.

Finally, Table 10 shows the calculated parameters for the complete formulation of the NASA9 polynomial which can be used to define the thermodynamic properties of each stoichiometric solid.

### 3.2. Validation of the Model: Heat Capacity Comparison and Vapor-Solid Equilibrium Curves

Once the fitting is successful with respect to their deviation parameters, the obtained parameters must be proved to check the goodness of the algorithm. The first checking procedure would be to compare the fitted heat capacity curve to an experimental output. For this, a sample of MgSO_4_·7H_2_O was measured in a Setaram BT 2.15 Calvet Calorimeter (Setaram – KEP Technologies, Caluire, France). The sample used for the experiment was obtained from Acros Organics and is of analysis grade with a purity of 99.5%. The water content of the substance was determined by Thermogravimetric Analysis to be 7.1 mol water per mol MgSO_4_. All heat capacity experiments were done by using the continuous cp measurement method with a heating rate of 0.3 K/min.

The Setaram BT2.15 Calorimeter uses a 3D-Calvet sensor (Setaram – KEP Technologies, Caluire, France) that surrounds the sample and was calibrated absolutely by Setaram with an electrical heater. The calorimeter is cooled with an external thermostat-controlled bath and permits a temperature range from −50 °C up to 200 °C. The volume of the sample cells amounts to 12 mL. A temperature calibration of the calorimeter has been done by measurements with different heating rates of Mercury, Gallium, and Indium with onset melting temperatures of −38.86 °C, 29.780 °C, and 156.5985 °C, respectively. The mean deviation between the measured onset temperatures (with calibration) and the appropriate reference values is 0.075 K, and all calibration experiments give onset melting temperatures within a range of ±0.22 K. The heat flow calibration of the calorimeter has been verified by cp measurements of NIST Standard Reference Material 720 (Synthetic Sapphire), where all results were within ±2% deviation compared to the reference values.

**Figure 18:**
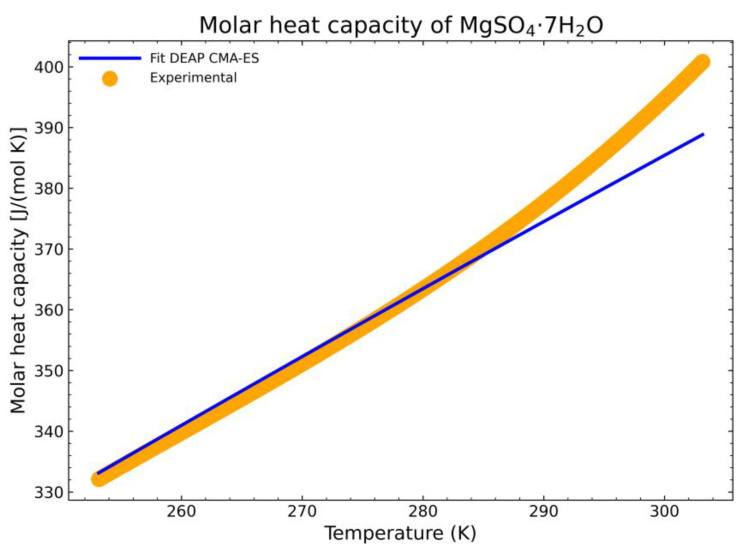
Comparison between the fitted heat capacity with the CMA-ES method and the experimental output from the DSC setup.

The selection of the heptahydrate form is done due to its phase stability at room temperature as it is the equilibrium phase at those conditions. The comparison is shown in Figure 18. The curve stops just before the phase transition to another binary mixture, in this case, it would transition to a mixture of an aqueous solution and the hexahydrate form [34]. This is shown by the rapid increase in slope at the end of the displayed experimental data. To obtain the heat capacity data after the phase transition, it would be necessary to calculate the phase equilibria first of the mixture of the solid hydrate and the aqueous solution with a suitable complex excess free energy model, which is beyond the scope of this work. The obtained residual standard deviation of the fitting is 3.974 J/(mol K) with respect to the experimental data. The estimation is in good agreement with the measured experimental data, calculated for *n* experimental points as:

*SD*_res_ = [Σ*_i_*(*c_p,i_*^mea^ − *c_p,i_*^fitted^)^2^/(*n* − 2)]^1/2^ (32)

To have a better overview of the deviation of the fitting procedure, the mean relative error (*MRE*) and the normalized root mean square deviation (*NRMSD*) were also calculated. The *MRE* is defined as:

*MRE* = 1/*n*[Σ*_i_*(|*c_p,i_*^mea^ − *c_p,i_*^fitted^|/*c_p,i_*^mea^)] (33)

which results in a *MRE* of 0.7%. The *NRMSD* is calculated as:

*NRMSD* = |[Σ*_i_*(*c_p,i_*^mea^ − *c_p,i_*^fitted^)^2^/*n*]^1/2^/[Σ*_i_*(*c_p,i_*^mea^)/*n*]| (34)

whose result is a *NRMSD* of 1.1%. Thus, the estimation is in good agreement with the measured experimental data.

The second comparison to validate the fitting is the construction of vapor-solid equilibrium curves. To perform the calculation, the Gibbs’ Free Energy of the possible phases involved must be first calculated. In this case, the energies of all possible hydrated phases from MgSO_4_ are calculated by means of the NASA9 parameters calculated in Table 10. For water, only solid and vapor phases are considered, as the liquid phase would need a more complicated formulation based on excess properties and it is beyond the scope of this work. For the solid water, a NASA7 polynomial from NIST [17,18] is employed. In the case of vapor, from the same reference data, another NASA7 polynomial is used to define the reference state. However, ideal gas formulation is used to calculate the thermodynamic properties of the vapor from the reference state point. To determine which phases are present at any given *T*–*p* combination and their phase transition, the VCS chemical stoichiometric solver [35] included in the Cantera open-source software [36], which allows its use in Python, is employed. The equilibrium curves between the different hydrate levels and the water vapor should give the dehydration curves. As a fixed initial composition is needed to perform the equilibrium calculations, the composition of salt and water corresponding to MgSO_4_·7H_2_O will be used as it is the most common stable form at standard conditions. It is worth noting that all possible hydrous forms are included in the calculations, i.e., if any of the hydrous compounds with its corresponding proposed stoichiometric model are much deviated from the real value at any temperature range, a different unexpected phase composition would appear in the computed diagram.

The calculation will be compared to two data sources: (1) experimental dehydration data from the heptahydrate and hexahydrate [37] as they are the most common stable forms at different temperature conditions by transforming RH data into pressure [38]; (2) the equilibrium curves of Van’t Hoff [39,40,41] from enthalpy and entropy of reaction/dehydration data of the following form:

ln(*p*/*p*^0^) = −Δ_r_*H*^0^/R*T* + Δ_r_*S*^0^/R, (35)

where *p*^0^ is the reference pressure or the atmospheric pressure (standard conditions) in this case. The enthalpy and entropy of reaction, Δ_r_*H*^0^ and Δ_r_*S*^0^, when the dehydration is seen as a reaction, can be calculated by the solvate difference rule [6]. This consists in the calculation of the enthalpy/entropy of reaction from the difference in enthalpy/entropy of formation between the reactants, corresponding to the hydrate before dehydration, and the products, formed by the lower-level hydrate and water vapor. The equation is defined in a per mol of water basis. In general, being A the anhydrous form and the dehydration occurring from an *n*th hydrate to an *m*th hydrate in the corresponding physical state, the enthalpy change of reaction can be defined as:

(*H*^0^_f,A*n*H_2_O(s)_)/(*n* − *m*) + Δ_r_*H*^0^ = (*H*^0^_f,A*m*H_2_O(s)_)/(*n* − *m*) + *H*^0^_f,H_2_O(g)_ (36)

(*S*^0^_f,A*n*H_2_O(s)_)/(*n* − *m*) + Δ_r_*S*^0^ = (*S*^0^_f,A*m*H_2_O(s)_)/(*n* − *m*) + *S*^0^_f,H_2_O(g)_ (37)

**Figure 19:**
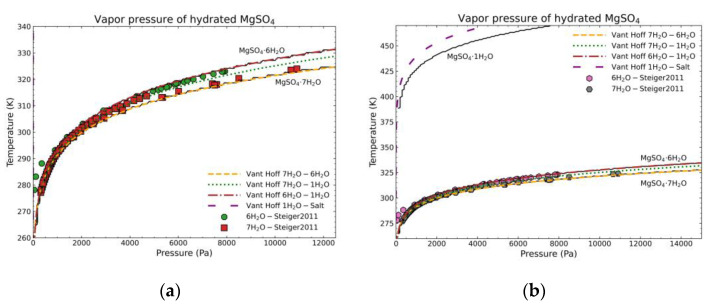
Temperature—vapor pressure comparison of MgSO_4_·7H_2_O between experimental data from Steiger et al. [37], Van’t Hoff curves, and the calculation of this work taking the MgSO_4_·7H_2_O as the fixed composition (black lines): (**a**) for a limited range to show literature points for the dehydration of MgSO_4_·7H_2_O and MgSO_4_·6H_2_O; (**b**) extended range to show further curves and the dehydration of MgSO_4_·1H_2_O.

Van’t Hoff curves are built with the standard formation data from [12] for the salt hydrates and [17] for vapor water. Figure 19 shows the results of the above calculations. The black solid lines represent the calculation done in this work, the points are the experimental data from the literature and the other lines are the corresponding Van’t Hoff equilibrium curves. Figure 19a shows the region where experimental data exist. In this case, the Van’t Hoff equilibrium curves for the dehydration of the heptahydrate and hexahydrate and the computed curves fit almost perfectly, while the experimental points are also showing almost the same behavior. Both are in good correlation with the algorithm computed data. Figure 19b shows also the dehydration of the monohydrate. In this case, no experimental data exist due to the difficulty of obtaining pure salt so the comparison can be only be performed between the calculation in this work and the Van’t Hoff curve. In this case, the trend is similar, the curve has a similar shape but there is a discrepancy of about 10 °C between them at any given pressure. As there is no experimental data available, it is difficult to justify which approach is better. However, both approaches indicate a similar dehydration behavior. As the determination of the vapor-solid equilibrium is dependent on the Gibbs’ Free Energy calculation, the result shows the goodness of the achieved enthalpy and entropy values fitted by the proposed thermodynamic model.

The two cases, where the proposed fitted model is employed, show good correlations with experimental data and other theoretical models. The CMA-ES method provides a new alternative to other methods with higher flexibility due to the definition of constraints, a more robust approach for data dispersion and a very high automation potential as it prevents manual tuning of every point by giving a weight factor depending on data type and source, providing still good results and avoiding subjective decisions about which sources to choose. The weight factors also provide a way of discarding data in the calculation with the flag “Not used” without removing them from the database as they may be needed in complicated data sets. The results indicate that the CMA-ES algorithm can be used not only for just heat capacity data, but also for more complex calculations like phase equilibria in case of stoichiometric solids.

## 4. Conclusions

The proposed constrained evolutionary strategy is a useful and easy way to implement an approach to fit thermodynamic data to any desired model, especially, when the dispersion of data is very large or when an automation of the fitting process is required. Furthermore, constraints can be added to force a correct physical or thermodynamic description which is an advantage over more commonly used fitting methods. A feasible thermodynamic description aids to choose the most relevant data in a more objective way, to improve the usability of such data in more complex processes, and to predict other derived properties such as the enthalpy, entropy, or Gibbs free energy from heat capacity data.

The algorithm is robust and stable. At the current state, the algorithm needs some manual tuning of certain parameters. However, most of the parameters need only one simulation beforehand, e.g., the penalty factors, and the benefits in the fitting procedure surpasses the disadvantages of the time consumed to tune the algorithm-specific coefficients.

The results show a reasonable agreement with the experimental comparison of the heat capacity and the vapor-solid equilibrium of the chosen family of hydrates for magnesium sulfate. This shows the goodness of the method to obtain thermodynamic data for the description of the solid, a stoichiometric solid’s Gibbs Free Energy model, and use this data consistently in more complex problems like the calculation of solid-vapor or solid-liquid phase equilibria where the calculated enthalpy and entropy values are critical.

The model does not pose any limitation for any kind of substance or fitted property when sufficient data is fed, especially, when physical constraints of behavior can be defined.

## Figures and Tables

**Table 1:** Gathered thermodynamic data for MgSO_4_·1H_2_O.

Type of Thermodynamic Data	Reference
Cp	Glasser, 2007 [6]
Cp, H^0^_f_, S^0^_f_	DeKock, 1986 [7]
Cp	Pabalan, 1987 [8]
Cp	Frost, 1957 [9]
Cp	Gmelin, 1939 [10]
Cp	Perry, 1999 [11]
H^0^_f_, S^0^_f_	Grevel, 2009 [12]
H^0^_f_, S^0^_f_	Wagman, 1982 [13]
H^0^_f_, S^0^_f_	Grevel, 2012 [14]
H^0^_f_, S^0^_f_	Dean, 1979 [15]
H^0^_f_, S^0^_f_	Billon, 2015 [16]

**Table 2:** Suggested weight factor for data pre-filtering.

Group	Level Description	Assigned Weight Factor
Data source	Level 0—Experimental	2^0^
	Level 1—Mixed	2^−1^
	Level 2—Table/Theoretical	2^−2^
	Level 3—Singular data	2^−3^
Equation type	Level 0—Point	2^0^
	Level 1—Quadratic	2^−1^
	Level 2—Linear	2^−2^
	Level 3—Constant	2^−3^

**Table 3:** Gathered thermodynamic data for MgSO_4._

Type of Thermodynamic Data	Reference
Cp	Glasser, 2007 [6]
Cp, H^0^_f_, S^0^_f_	DeKock, 1986 [7]
Cp	Pabalan, 1987 [8]
Cp, H^0^_f_, S^0^_f_	Perry, 1999 [11]
H^0^_f_, S^0^_f_	Grevel, 2009 [12]
Cp, H^0^_f_, S^0^_f_	Wagman, 1982 [13]
Cp, H^0^_f_, S^0^_f_	Dean, 1979 [15]
H^0^_f_, S^0^_f_	Billon, 2015 [16]
Cp, H^0^_f_, S^0^_f_	Aylward, 1975 [20]
Cp, H^0^_f_, S^0^_f_	Robie, 1995 [21]
Cp, H^0^_f_, S^0^_f_	CRC_Handbook, 2003 [22]
Cp, H^0^_f_, S^0^_f_	Patnaik, 2003 [23]
H^0^_f_, S^0^_f_	Rayner, 2010 [24]

**Table 4:** Gathered thermodynamic data for MgSO_4_·2H_2_O, MgSO_4_·4H_2_O, MgSO_4_·5H_2_O, MgSO_4_·6H_2_O, and MgSO_4_·7H_2_O.

Type of Thermodynamic Data	Reference	Substance
Cp	Glasser, 2007 [6]	MgSO_4_·2H_2_O, MgSO_4_·4H_2_O, MgSO_4_·6H_2_O, MgSO_4_·7H_2_O
Cp, H^0^_f_, S^0^_f_	DeKock, 1986 [7]	MgSO_4_·2H_2_O, MgSO_4_·4H_2_O, MgSO_4_·6H_2_O, MgSO_4_·7H_2_O
Cp, H^0^_f_, S^0^_f_	Pabalan, 1987 [8]	MgSO_4_·5H_2_O, MgSO_4_·6H_2_O, MgSO_4_·7H_2_O
Cp	Gmelin, 1939 [10]	MgSO_4_·6H_2_O, MgSO_4_·7H_2_O
Cp	Perry, 1999 [11]	MgSO_4_·6H_2_O, MgSO_4_·7H_2_O
Cp, H^0^_f_, S^0^_f_	Grevel, 2009 [12]	MgSO_4_·2H_2_O, MgSO_4_·4H_2_O, MgSO_4_·6H_2_O, MgSO_4_·7H_2_O
Cp, H^0^_f_, S^0^_f_	Wagman, 1982 [13]	MgSO_4_·2H_2_O, MgSO_4_·4H_2_O, MgSO_4_·6H_2_O, MgSO_4_·7H_2_O
H^0^_f_, S^0^_f_	Grevel, 2012 [14]	MgSO_4_·4H_2_O, MgSO_4_·6H_2_O, MgSO_4_·7H_2_O
H^0^_f_, S^0^_f_	Dean, 1979 [15]	MgSO_4_·7H_2_O
H^0^_f_, S^0^_f_	Billon, 2015 [16]	MgSO_4_·4H_2_O, MgSO_4_·5H_2_O, MgSO_4_·6H_2_O, MgSO_4_·7H_2_O
H^0^_f_, S^0^_f_	Aylward, 1975 [20]	MgSO_4_·7H_2_O
H^0^_f_, S^0^_f_	Robie, 1995 [21]	MgSO_4_·7H_2_O
Cp, H^0^_f_, S^0^_f_	Patnaik, 2003 [23]	MgSO_4_·2H_2_O, MgSO_4_·4H_2_O, MgSO_4_·6H_2_O, MgSO_4_·7H_2_O
H^0^_f_, S^0^_f_	Rayner, 2010 [24]	MgSO_4_·7H_2_O
Cp, S^0^_f_	Cox, 1955 [32]	MgSO_4_·6H_2_O
Cp, S^0^_f_	Gurevich, 2007 [33]	MgSO_4_·7H_2_O

**Table 5:** Penalty factors for the simulation.

Minimum Initial Residual (No pty)	Maximum Final Residual (No pty)	pty_0_	pty_1_	pty_2_	pty_3_
10^16^	10^2^	10^6^	10^12^	10^4^	10^9^

**Table 6:** Parameters of the simulation for the fitting of the heat capacities and final residual of the calculation.

Substance	Iterations	Population (λ)	Initial Distribution (σ)	Residual
MgSO_4_	290	315	3	1.72 × 10^2^
MgSO_4_·1H_2_O	280	315	3	3.17 × 10^1^
MgSO_4_·2H_2_O	350	315	3	2.75 × 10^−4^
MgSO_4_·4H_2_O	350	315	3	4.26 × 10^3^
MgSO_4_·5H_2_O	350	315	3	6.11 × 10^−26^
MgSO_4_·6H_2_O	350	315	3	1.50 × 10^3^
MgSO_4_·7H_2_O	350	315	3	5.79 × 10^3^

**Table 7:** Statistical error and deviations of the fitted heat capacity with different fitting methods, *n* is the number of compared points or population.

	*MRE*			*NRMSD*				
Substance	LM	LM + Weight Factor	CMA-ES	LM	LM + Weight Factor	CMA-ES	*n*	Number of Sources
MgSO_4_	3.02%	2.98%	3.92%	3.66%	4.73%	5.11%	694	10
MgSO_4_·1H_2_O	2.49%	1.94%	2.06%	3.55%	4.09%	4.34%	438	7
MgSO_4_·2H_2_O	0.02%	0.02%	0.02%	0.02%	0.03%	0.03%	13	2
MgSO_4_·4H_2_O	70.85%	51.21%	278.4%	2.15%	2.19%	6.63%	134	4
MgSO_4_·5H_2_O	<0.01%	<0.01%	<0.01%	<0.01%	<0.01%	<0.01%	201	1
MgSO_4_·6H_2_O	1.02%	0.87%	2.21%	1.06%	1.18%	1.39%	421	10
MgSO_4_·7H_2_O	246.79%	468.28%	24.93%	2.88%	3.04%	3.09%	650	9

**Table 8:** Parameters of the simulation for the fitting of enthalpy and entropy.

Substance	Iterations	Population (λ)	Initial Distribution (σ)
All	100	55	5

**Table 9:** Statistical error and deviations of the fitted enthalpy and entropy with the CMA-ES method, *n* is the number of compared points or population.

	Enthalpy			Entropy		
Substance	*MRE*	*NRMSD*	*n*	*MRE*	*NRMSD*	*n*
MgSO_4_	0.81%	1.85%	15	0.54%	1.41%	14
MgSO_4_·1H_2_O	0.21%	0.29%	10	1.80%	3.93%	8
MgSO_4_·2H_2_O	0.03%	0.04%	4	10.22%	11.17%	2
MgSO_4_·4H_2_O	0.03%	0.04%	9	1.71%	1.88%	6
MgSO_4_·5H_2_O	0.03%	0.03%	2	1.65%	1.65%	2
MgSO_4_·6H_2_O	0.03%	0.04%	10	0.12%	0.28%	9
MgSO_4_·7H_2_O	0.02%	0.03%	14	0.60%	1.28%	14

**Table 10:** Calculated NASA9 parameters through the CMA-ES method.

Substance	a_0_	a_1_	a_2_	a_3_	a_4_	a_5_	a_6_	a_7_	a_8_
MgSO_4_	2.41 × 10^−2^	−9.67 × 10^−3^	2.69 × 10^0^	3.86 × 10^−2^	−3.86 × 10^−5^	1.93 × 10^−8^	−3.62 × 10^−12^	−1.57 × 10^5^	−1.43 × 10^1^
MgSO_4_·1H_2_O	2.37 × 10^−2^	−1.38 × 10^−2^	6.22 × 10^0^	3.36 × 10^−2^	−3.19 × 10^−6^	4.44 × 10^−9^	−2.31 × 10^−12^	−1.97 × 10^5^	−3.01 × 10^1^
MgSO_4_·2H_2_O	3.77 × 10^−2^	−2.15 × 10^−2^	5.61 × 10^0^	5.39 × 10^−2^	−9.19 × 10^−6^	1.04 × 10^−8^	−4.45 × 10^−2^	−2.32 × 10^5^	−2.83 × 10^1^
MgSO_4_·4H_2_O	−1.24 × 10^−1^	3.71 × 10^−1^	−3.71 × 10^−1^	1.24 × 10^−1^	−5.20 × 10^−5^	3.78 × 10^−9^	1.22 × 10^−12^	−3.05 × 10^5^	−2.21 × 10^0^
MgSO_4_·5H_2_O	3.51 × 10^−9^	−1.18 × 10^−9^	1.02 × 10^1^	8.93 × 10^−2^	−2.09 × 10^−17^	1.37 × 10^−19^	−1.34 × 10^−22^	−3.43 × 10^5^	−4.84 × 10^1^
MgSO_4_·6H_2_O	−1.76 × 10^−1^	5.27 × 10^−1^	−5.28 × 10^−1^	1.76 × 10^−1^	−1.19 × 10^−4^	3.54 × 10^−8^	−3.88 × 10^−12^	−3.78 × 10^5^	−2.64 × 10^0^
MgSO_4_·7H_2_O	−1.84 × 10^−1^	5.51 × 10^−1^	−5.51 × 10^−1^	1.84 × 10^−1^	−9.80 × 10^−5^	1.93 × 10^−8^	−7.53 × 10^−13^	−4.15 × 10^5^	−2.71 × 10^0^

## Data Availability

The data presented in this study are available on request from the corresponding author.
